# The cost-saving effect of continuity of care (COC): an analysis of institutional COC on diabetic treatment costs using panel 2SLS regressions

**DOI:** 10.1186/s12913-021-07052-5

**Published:** 2021-10-18

**Authors:** Yu-Ping Wen, Sandra S. Liu, Ji-Tian Sheu, Hong-Huei Wang, Edmund R. Becker, Jui-fen Rachel Lu

**Affiliations:** 1 Department of Health Care Management, College of Management, Chang Gung University, Taoyuan, Taiwan, R. O. C.; 2 Professor Emerita in Public Health, Purdue University, West Lafayette, Indiana USA; 3 Department of Nursing, Linkou Branch, Chang Gung Memorial Hospital, Taoyuan, Taiwan, R. O. C.; 4 Rollins School of Public Health, Emory University, Atlanta, Georgia USA; 5 Graduate Institute of Management, College of Management, Chang Gung University, Taoyuan, Taiwan, R. O. C.; 6 Department of Radiation Oncology, Linkou Branch, Chang Gung Memorial Hospital, Taoyuan, Taiwan, R. O. C.

**Keywords:** Continuity of care, Diabetes mellitus, Team care, Two-stage least squares regression, Costs

## Abstract

**Background:**

The high costs of chronic conditions call for new treatment approaches that reduce costs while ensuring desirable health outcomes. There has been a growing transformation of care delivery models from conventional referral systems to integrated care models. This study seeks to evaluate the cost-saving impact of integrated care delivery model under pay-for-performance (P4P) scheme with continuity of care at institution level (ICOC).

**Methods:**

We analyzed the Taiwan National Health Insurance claim data of 21,725 diabetic patients who visited clinics and/or hospitals at least four times a year for 8 years. Using average local provider P4P participation rate (for each accreditation level) as an instrumental variable in two-stage least squares (2SLS) regressions, we have estimated consistent estimates of the ICOC elasticities for all-cause inpatient and outpatient costs.

**Results:**

Our results show that ICOC significantly reduced inpatient costs but increased outpatient costs with the elasticity for treatment costs of -11.6 and 1.03, respectively. The decrease in inpatient costs offset the increase in outpatient costs and the resulting total cost saving showed significant association with ICOC. The saving effect of ICOC is especially robust among patients who used clinics as their principal source of care.

**Conclusions:**

Institutional continuity of care has a substantial impact on the treatment costs of diabetes patients. In the context where inpatient care costs are significantly higher than that of the outpatient care, ICOC would lead to a meaningful cost-saving effect. For new diabetes patients, care by clinics demonstrated the strongest saving effect.

## Background

In 2019, approximately 463 million adults (20–79 years) were living with diabetes. By 2045, the International Diabetes Federation (IDF) estimates this number will rise to 700 million adults; a 51 % increase [1]. The Global Burden of Disease Study 2017 ranked diabetes as the fourth leading cause of age-standardized years lived-with-disability in 2017, and the Global Burden of Disease Study 2019 ranked diabetes as the number eighth leading cause of all-age burden of disease (disability-adjusted life-years) in 2019 [2, 3]. The high economic and social costs of diabetes and its rising prevalence, especially the Type 2 diabetes where most adults eventually need to get insulin injections, make a strong case for interventions that improve both the quality of outcomes and costs for diabetes patients.

Considerable efforts have been made to improve the health care delivery system for chronic care in recent decades. There has been a shift of care delivery from the primary physician care to integrated team-care with the goals of improving fragmented care, improving outcomes, and using resources more efficiently. Sometimes these efforts have involved structural changes [4]. Prominent among these structural changes have been the growth of patient-centered medical homes (PCMHs) and accountable care organizations (ACOs) in the US [5, 6]. These models involve a broader array of health professionals besides physicians, such as nurses and care coordinators, and coordinating care across settings, institutions, and medical groups. A central feature of these care models is integrating multiple facets of care for diabetes patients. However, the composition and organization of the systems and the degree and extent of care integration varies greatly and, consequently, the effectiveness of the care models is mixed [7–9]. In addition, many formally independent primary care clinics in the US are becoming part of hospital or integrated health care system [10] and the new care models are raising concerns regarding the roles and survival of small primary practices [11, 12].

“Continuity of care” (COC) has been a central concept for primary care [13]. As the care for chronic diseases moves away from single-physician primary care, there is a gap between the conventional wisdom of physician-based continuity of care and these new models of care [14]. Conventionally, seeing the same physician for care is considered to have stability and continuity in care on three perspectives: relational, informational, and management [15]. When care models shift beyond single physician-level to teams or medical groups, the relational continuity at physician level is lowered. As a result, the impact and change in information and management perspectives and the continuity in these perspectives depends on the structural organization and systems of the institutions involved [4]. Moreover, it raises an important question, that is, how to measure COC under new models of care when physicians have many partners to provide care?

This study explores COC from an institutional point of view. We propose a new measure of care continuity, the institutional COC (ICOC) to reflect the institution/system-based care. The ICOC is straightforward to calculate (as shown in our methods section below) and typically serves as a measure of care concentration within medical systems. In fact, some previous studies have already measured continuity at the site-level. For example, Flach et al. used 4 items to measure continuity of care at the Veterans Health Administration hospitals at the facility-level [16]. Guo et al. used the hospital as the observing unit for COC [17]. However, the effectiveness of ICOC on treatment costs is yet to be examined and demonstrated.

In the literature, the physician-level COC has shown cost-saving effects, typically for preventing hospitalizations and emergency use. Other studies found that COC reduced DM inpatient utilization approximately 15–20 % for patients with high COC, or improved patient health outcome such as reduction in mortality (8.6 % vs. 18.5 %) [18–20]. Lubloy et al. showed that stronger GP specialist connections resulted in lower pharmacy costs, but not in better health outcomes [21]. Thus, the cost-saving effects of COC were conjectured to be due to better information sharing, improved interpersonal relationships, improved continuity in management, and better medication adherence [18, 22, 23].

However, previous studies failed to consider the endogeneity issue related to COC, hence, the true cost-saving effect of COC has been understudied. Only Pu et al. used the average diabetes-specific COC scores within families as an instrumental variable to control endogeneity to estimate COC’s impact on the emergency room use in the following year. They concluded that the marginal effect of COC on reducing the probability of emergency room use actually increased after endogeneity of COC was considered [24]. However, they did not analyze whether reduction in emergency use resulted in saving costs.

Thus, our study aims to take advantage of multi-year national diabetes data to test how COC at the institution level is associated with treatment costs using the ICOC as a measure of COC for institution/system-based care. In order to achieve the research goal, we extended the observation period to 8 years and adopted a panel two-stage least squares regression (2SLS) modeling framework to produce consistent cost-saving estimates for ICOC for both all-cause outpatient and inpatient care. In the process, we simulate the common practice of using COC in the last period to predict costs in the next period by lagging ICOC 1 to 3 years.

## Methods

### Data sources and sample

The data employed for analyses were extracted from Taiwan National Health Insurance (NHI) Diabetes Database. The NHI claim data used in this study were compiled and encrypted by Taiwan National Health Insurance Administration, and approved and released for academic research by Taiwan National Health Research Institutes. The selected patients were newly diagnosed patients with DM (had no DM-related cost record in 2000) and had at least two visits with a principal ICD-9-CM diagnoses code of DM in 2001. As the Pay-for-Performance (P4P) program was implemented in 2001, patients newly diagnosed of DM in 2001 would be subject to the P4P policy. As the timing of DM diagnoses could vary among patients in 2001, year 2002 was set as baseline to establish a consistent and uniform 8-year observation period.

Other inclusion criteria include patients aged 25 years or older and holding a valid insurance enrollment status through the study period from 2002 to 2009. Those with gestational diabetes or were deceased during the study period were excluded. The ICOC score was calculated from those patients who visited their respective providers at least 4 times per year to allow the comparability of our study with other COC studies [25]. The final analytical sample size was 21,725 patients.

### Institutional characteristics of the DM treatment in Taiwan

Taiwan’s NHI program is a single-payer system, which covers over 99 % of the population and contracts with 93 % of medical institutions in Taiwan. Almost all the hospitals in Taiwan have a closed-staff feature, that is, most admissions are referred from their own outpatient departments. Patients enjoy full freedom to choose physicians and institutions for medical care and there is no gate-keeping mechanism [24]. The average annual per capita outpatient visits was 15.1 times (including visits to dentist and Chinese medicine doctor) and 0.14 hospital admission in 2018 [26].

The NHI payment scheme varies by the provider’s accreditation level. On average, it costed US$125 for an outpatient visit at medical center, and US$27 at clinics. The average claim cost per hospitalization in 2017 was US$2,800, $1,950, and $1,800 for medical centers, regional hospitals, and community hospitals, respectively. The average expenditure incurred per hospital day was around US$200 [26].

The prevalence of diabetes among Taiwanese adults was estimated to be 10.9 % [27]. Diabetes accounted for 5.1 % of total NHI outpatient expenditures. To improve the quality and efficiency of DM care, Taiwan NHI scheme has implemented a Pay-for-Performance (P4P) program which incentivize those enrolled institutions and physicians with a higher reimbursement rate since 2001. The program also required that the participating physicians be supported by a team of nurses, health promotion specialists, and nutritionists. Under the Taiwan NHI system, the DM patients are either diagnosed and prescribed with medication by primary care providers or endocrinologists. Check-ups and follow-up testing performed within the same institution regardless of prescribing physicians were all counted as part of the P4P performance [28]. There were no specific guidelines from NHI for care coordination.

The integrated care team model could also have an effect beyond P4P participating physicians and patients. Patient P4P enrollment was voluntary upon the physician’s invitation. Case managers of the P4P program would call patients to make sure they got regular check-ups. The nutritionists typically provided counseling to both P4P participants and non-P4P patients. Participating patients would have free access to health and dietary information sessions which otherwise charges a fee.

The NHI program reimburses the participating institutions instead of the individual physicians. The institutions then distribute physician fees to the participating clinicians based on internally determined formula and allocate additional case-management fees for diabetes-related team-care. About 7 % of the NHI contracted institutions participated in P4P [28]. Consequently, the institution-based P4P team-care provides a unique opportunity to investigate how ICOC is associated with treatment costs.

### ICOC Elasticity for Treatment Costs Estimation

The empirical panel fixed-effect OLS model for medical costs is indicated as Eq. (1)

1 $${lny}_{i, t}= {\alpha }_{0}+\beta p{t}_{i, t}+\mu pvd{r}_{i, t}+\delta P4{P}_{i, t}+\gamma lnICO{C}_{i,t}+{t}_{t}+{\alpha }_{i}+{\epsilon }_{i, t}$$

specifying that treatment costs y_i,t_ of patient i during year t is associated with patient characteristics vector, pt_i,t_ (such as diabetes severity); principal provider characteristics vector, pvdr_i,t_; patient participation in P4P program, P4P_i,t_; lnICOC_i,t_ taking natural logarithm of the ICOC score, patient specific error term, α_i_, and random errors, ε_i,t_. All costs are in natural logarithm to approximate normal distribution. As our sample is composed of patients who visited their respective providers at least four times per year and the annual average probability of inpatient use is around 15 %, the inpatient cost might contain value of zero. To avoid taking log of zero, $1 was added to inpatient cost, which would generate a value of 0 by this log transformation. This $1 addition practice is a common method in coping with expenditures (with zero value) in logarithm form and applies only to our inpatient cost model [29].

A patient was defined as a P4P participant if any NHI P4P claim codes were identified during year t. The higher treatment costs related to P4P payment system are also controlled by the P4P variable. DM severity was measured by Diabetes Complications Severity Index scores (DCSI), which was developed by Selby et al. [30]. The DCSI ranges from 0 to 13 with a higher score indicating a higher level of severity. The score was calculated cumulatively for each patient based on diagnoses recorded in outpatient and inpatient claims per annum, which includes 7 major comorbidities of diabetes: cardiovascular disease, nephropathy, retinopathy, peripheral vascular disease, stroke, neuropathy, and metabolism.

The provider characteristics pvdr_i,t_ depicted the institution where patient i seeks medical care at time t. If a patient visited more than one institution during the same year, the most-visited institution was assigned as the principal provider. Provider characteristics included ownership, NHI accreditation level, and location. A set of time dummy variables for year 2003 through 2009 were included to control for common trend in policies and ageing.

The ICOC score used in this study was commonly termed as the “continuity of care index”, as shown in Eq. (2) [25]:

2 $$\left[\right({\sum }_{j=1}^{M}{n}_{i,j, t}^{2 })-{N}_{i,t}]/\left[{N}_{i, t}\right({N}_{i, t}-1\left)\right]$$

where N_i,t_ is the total number of outpatient visits made by patient i during year t. The number of visits to institution j is denoted as n_i, j, t_. The ICOC score ranges between 0 and 1, with 1 indicating the highest degree of continuity. The ICOC score for the same number of visits varies according to the number of institutions that provided care to the patient. More institutions involved in providing the patient care, the lower the ICOC score would be for the same total number of visits. Appendix 1 illustrates how ICOC score changes for patients with different number of visits and number of institutions visited. One additional visit results in various degree of change in ICOC depending on the original number and distribution of visits. All ICOC scores are in natural logarithm in the regressions. The estimated coefficients γfor ICOC indicate the percentage of changes in costs associating with 1 % change in ICOC score. These coefficients reflect the ICOC elasticities for treatment costs. Based on previous studies, we hypothesize that the ICOC elasticities for inpatient costs are negative.

To consistently estimate the cost-saving effect of ICOC, we need to address the potential endogeneity of ICOC. While COC is mainly affected by personal traits and interaction with physician, ICOC is further affected by patient health and provider characteristics [31]. For example, as disease progresses, patients may need additional expensive specialist care. Gulliford et al. showed that, as the severity level of DM increased, patients’ COC declined [22]. How ICOC changes with this progression of disease, will depend on the provider’s specialty mix. If the patient used only one provider and could find a specialist needed within the same provider institution, the COC would decline without a change in ICOC, and treatment costs would increase. By contrast, additional outpatient visits outside the principal provider would lead to declines in both COC and ICOC, and an increase in health care costs. A negative correlation between ICOC and treatment costs due to health change may lead to an over-estimate of the saving effect of ICOC.

Another reason that ICOC may be endogenous is a result of Taiwan’s NHI payment design. Without formal primary care physician (family doctor) arrangement, patients can self-select themselves into different types of principal providers. Typically, patients with more severe conditions prefer medical centers whereas those with minor conditions would choose clinics as their principal providers [32]. NHI reimburses providers according to their accreditation levels. When medical centers have sufficient specialty mix, patients’ poor health is linked to high ICOC and high costs. On the other hand, healthy patients using small clinics will experience high ICOC and low costs. The value of ICOC is affected by provider scale and specialty mix and its effect on costs becomes ambiguous.

Therefore, this study employed the commonly used 2SLS method to address the endogeneity problem. The model involves two stages of regressions to provide consistent coefficient estimates for variables with endogeneity. In the first stage, an instrumental variable (IV) is chosen to predict individual ICOC scores. The criterion for choosing an IV is that the variable is closely related to ICOC but exogenous to the dependent variable, i.e., the treatment costs, so the IV will not correlate with the error terms Ɛ_i, t_. All variables in Eq. (1) and the IV are used to predict the ICOC score for each patient. At stage two, the estimated ICOC instead of the actual ICOC scores, will be used in regression models to ensure obtaining consistent estimates for the elasticities γ [33].

In the first stage, we chose the “average local provider P4P participation rate (for each accreditation-level)” as the IV. Unlike the family member COC which Pu et al. chose as the IV for patient COC, ICOC in this study is much more closely related to provider characteristics [24]. The average local provider P4P participation rate reflects the prevalence of P4P teams in the local area. Consequently, we used the entire national diabetes dataset, not just our new DM patient analytical sample, to calculate the local P4P participation rate for all 369 townships nation-wide. Next, we calculated the average provider P4P participation rate for each accreditation level within a township. The average local provider P4P participation rate was assigned to patients as the IV for ICOC according to the accreditation level and location of patients’ principal providers. We use software Stata package xtivreg2 for 2SLS regression analyses, which implements estimation of panel data models with potential endogenous variable and provides statistics for testing the weakness and endogeneity of the IV.

We began analyses with panel OLS to examine how changes in the values of variables might affect the treatment costs. Hausman tests were used to determine whether patient-specific costs were random or fixed. Then we focused on the 2SLS estimates for the cost-saving effect of ICOC, and compared with OLS results. We allowed the ICOC variables in the regressions to lag by 1 to 3 years to mimic the common practice of using COC at baseline to predict the cost effect in the following years. Finally, we used subsamples of patients who patron clinics as principal care provider to re-estimate the ICOC elasticities for treatment costs to investigate how ICOC elasticities change with provider characteristics.

## Results

### Sample Characteristics

The patient characteristics, as shown in Table 1, reflect that patients self-selected themselves into different types of providers. Patients were grouped in accordance with the accreditation level of their principal providers in 2002 and 2009, respectively. As shown by the data, patients frequently changed their principal providers from year to year. The ICOC and COC scores varied significantly across providers. Those patients who chose clinics as principal providers were more likely to have the highest COC but lowest ICOC. Patient health (DCSI scores), age, and participation in P4P program were also significantly different among providers. Those patients who were cared by the clinics tended to be healthier and they were more likely to be women.

**Table 1:** Sample characteristics by principal provider type (*n*=21,725)

	Medical center		Regional hospitals		Community hospitals		Clinics	
**Patient characteristics**	2002	2009	2002	2009	2002	2009	2002	2009
	*N*=5,319	*N*=5,026	*N*=5,623	*N*=6,096	*N*=4,144	3,541	*N*=6,637	*N*=7,062
ICOC	0.85 (0.22)	0.90 (0.19)	0.85 (0.23)	0.88 (0.20)	0.82 (0.25)	0.84 (0.22)	0.81 (0.64)	0.84 (0.23)
COC	0.64 (0.30)	0.71 (0.29)	0.64 (0.30)	0.70 (0.28)	0.58 (0.31)	0.66 (0.30)	0.71 (0.30)	0.72 (0.28)
DCSI score	0.64 (1.05)	2.08 (2.00)	0.61 (1.00)	2.13 (1.98)	0.58 (0.95)	2.15 (1.98)	0.31 (0.67)	1.56 (1.64)
Sex (female=1)	0.44 (0.50)	0.44 (0.50)	0.47 (0.50)	0.46 (0.50)	0.48 (0.50)	0.47 (0.50)	0.51 (0.50)	0.51 (0.50)
Age	56.36 (11.66)	63.91 (11.57)	56.49 (11.66)	63.95 (11.68)	57.59 (11.66)	64.51 (11.86)	57.41 (11.08)	63.62 (11.04)
Participated in P4P	0.06 (0.24)	0.20 (0.40)	0.21 (0.40)	0.35 (0.48)	0.07 (0.26)	0.26 (0.44)	0.03 (0.18)	0.17 (0.38)
**Provider Ownership**								
Public	0.26 (0.44)	0.28 (0.45)	0.34 (0.46)	0.31 (0.46)	0.15 (0.36)	0.17 (0.38)	0.18 (0.38)	0.16 (0.36)
For-profit	0.15 (0.36)	0.17 (0.37)	0.24 (0.43)	0.10(0.30)	0.74 (0.44	0.60 (0.49)	0.82 (0.39)	0.82 (0.38)
Non-profit	0.57 (0.49)	0.55 (0.50)	0.41 (0.49)	0.59 (0.49)	0.11 (0.31)	0.23 (0.42)	0.00 (0.05)	0.02 (0.14)
**Local Provider P4Pparticipating rate **	0.53 (0.50)	0.99 (0.02)	0.57 (0.44)	0.97 (0.08)	0.15 (0.25)	0.44 (0.32)	0.01 (0.04)	0.05 (0.08)
**Outpatient cost**								
Total No. of visits	10.81 (5050)	16.10 (7.89)	9.88 (5.00)	15.58 (7.01)	9.52 (5.01)	15.56 (8.0)	9.43 (4.43)	12.42 (6.16)
OP costs (NTD)	13,223 (12,263)	31,533 (40,683)	11,540 (9,378)	29,626 (34,910)	9,768 (7,581)	26,546 (20,475)	6,481 (5,328)	14,268 (13,790)
**Inpatient cost**								
No of admissions	0.45 (0.89)	0.33 (0.89)	0.40 (0.87)	0.39 (1.0)	0.34 (0.82)	0.35 (1.04)	0.12 (0.45)	0.18 (0.64)
LOS	4.39 (16.87)	3.04 (11.30)	3.32 (12.9)	3.24 (11.46)	2.64 (12.77)	3.21 (13.04)	0.86 (5.38)	1.23 (6.32)
IP costs (NTD)	21,390 (71,724)	24,378 (97,956)	13,339 (48,424)	21,306 (77,599)	9,600 (35,990)	18,135 (71,396)	4,328 (31,358)	9,538 (51,642)
**Total costs (NTD)**	34,614 (74,896)	55,912 (110,233)	24,879 (50,579)	50,932 (88,017)	19,368 (37,906)	44,618 (75,462)	10,810 (32,069)	23,806 (54,303)

Note: Figures in parentheses are SD; NTD: New Taiwan Dollars, local currency in Taiwan.

All variables were significantly different at 0.01 for 2002 and 2009.

The conventional thinking is that patients may need to visit larger hospitals as their conditions progress, and they need more specialist care. The patients in this study, however, by 2009, tended to use clinics more as their principal providers, perhaps because during this time Taiwan was experiencing an increasing number of small-scale for-profit hospitals closure while more clinics were entering the market. Also, during this period, only 24 % of clinics participated in P4P program but almost all medical centers and regional hospitals had some physician-led teams that have joined P4P.

As for the treatment costs, patients using clinics as their principal providers incurred the least costs while patients with the regional hospitals or medical centers at higher accreditation level incurred higher treatment costs. For all providers, the average outpatient costs, not surprisingly, were approximately half of the inpatient costs. Annual numbers of admissions were as low as 0.18 for patients cared at clinics in 2009.

### ICOC elasticity: Panel OLS vs. 2SLS estimates

Table 2 reports the regression estimates for ICOC elasticities. When using panel ordinary least squares (OLS), the ICOC was negatively and significantly related to all types of treatment costs, namely − 0.07, -1.71, and − 0.3 for outpatient, inpatient, and total costs, respectively. For 2SLS, the first stage regression results (not reported in the tables) revealed that average local provider P4P participation rate (by accreditation-level) was positively and significantly associated with patient ICOC. The second stage regressions produced a different ICOC elasticity for outpatient costs, i.e., 1.03 and a change of sign. This indicates that a 1 % increase in ICOC was associated with 1 % increase in outpatient costs. The 2SLS ICOC elasticity for inpatient care was − 11.6, greater than the OLS estimate, -1.71.

**Table 2:** Panel OLS and 2SLS regression estimates

OLS model	OP costs		IP costs		Total costs	
ln ICOC	-0.07 (0.005)	*	-1.71 (0.03)	**	-0.30 (0.006)	**
DCSI score	0.21 (0.002)	*	0.44 (0.01)	*	0.25 (0.002)	*
P4P	0.33 (0.005)	*	-0.27 (0.03)	*	0.26 (0.01)	*
Accreditation level (ref = community hospital)						
Medical center	0.07 (0.01)	*	0.64 (0.06)	*	0.16 (0.01)	*
Regional hospital	0.08 (0.01)	*	0.49 (0.05)	*	0.12 (0.01)	*
Clinics	-0.40 (0.01)	*	-0.68 (0.05)	*	-0.45 (0.01)	*
Ownership (ref = for-profit)						
Public	0.12 (0.01)	*	0.004 (0.05)		0.12 (0.01)	*
Non-profit	0.17 (0.01)	*	-0.11 (0.05)	*	0.16 (0.01)	*
constant	9.18 (0.01)	*	0.86 (0.04)	*	9.31 (0.01)	*
rho	0.50		0.20		0.39	
Overall R^2^	0.25		0.10		0.26	
**2SLS model**						
ln ICOC	1.03 (0.26)	*	-11.62 (1.93)	**	-1.07 (0.31)	**
DCSI score	0.12 (0.01)	*	0.30 (0.07)	*	0.12 (0.01)	*
P4P	0.26 (0.01)	*	-0.40 (0.07)	*	0.16 (0.01)	*
Accreditation level (ref = community hospital)						
Medical center	0.09 (0.01)	*	0.81 (0.09)	*	0.21 (0.01)	*
Regional hospital	0.05 (0.01)	*	0.85 (0.10)	*	0.16 (0.02)	*
Clinics	-0.47 (0.01)	*	-0.59 (0.06)	*	-0.51 (0.01)	*
Ownership (ref = for-profit)						
Public	0.08 (0.01)	*	0.17 (0.07)	*	0.11 (0.01)	*
Non-profit	0.11 (0.01)	*	-0.03 (0.06)		0.101 (0.01)	*
F (15,152049)	3335	*	238	*	2283	*
Tests of instruments						
Weak Instrument (Anderson-Rubin Wald test F)	23.3	*	61.46	*	12.54	*
Wald test for endogeneity χ^2^(1)	30.56	*	46.68	*	5.75	†

Note: *: *p*-value < 0.01; †: *p*-value < 0.05

The Andersen-Rubin Wald F statistics rejected the hypothesis that average local provider P4P participation rate was a weak instrument, and the Wald test for endogeneity supported that ICOC was endogenous for both outpatient and inpatient costs. Though ICOC illustrated opposite effects in outpatient and inpatient costs, ICOC elasticity for total costs was − 1.07 and reached statistical significance.

Table 3 delineates the findings from investigating the impact of the lagged ICOC on costs with 2SLS regressions from year 1 to 3. By using the lagged values of ICOC (for OLS) or estimated ICOC (for 2SLS), the number of observations that could be used for estimation decreased with the number of years lagged. When ICOC was lagged by 1 year, the signs of elasticity remained unchanged as when using concurrent ICOC, but the magnitude of elasticities increased. The ICOC elasticities for inpatient and total costs were consistently negative, but the sign for the ICOC elasticity on outpatient costs changed with the number of years lagged.

**Table 3:** ICOC elasticity for treatment cost estimates with 1–3 years lagged

		ICOC elasticity					
		OP costs		IP costs		Total costs	
**OLS model**	lag 1 year	-0.02 (0.004)	*	0.08 (0.03)	**	-0.01 (0.006)	*†
	lag 2 years	0.01 (0.005)	†	0.23 (0.03)	*	0.04 (0.01)	*
	lag 3 years	0.03 (0.005)	*	0.22 (0.04)	*	0.06 (0.007)	*
**2SLS model**	lag1 year	1.07 (0.21)	*	-23.32 (2.83)	*	-1.93 (0.32)	*
	Weak Instrument (Anderson-Rubin Wald test F)	37.72	*	351.86	*	65.52	*
	Wald test for endogeneity χ^2^(1)	39.51	*	353.67	*	61.78	*
	lag 2 years	-0.34 (0.18)		-12.07 (1.94)	*	-1.94 (0.34)	*
	Weak Instrument (Anderson-Rubin Wald test F)	3.85	†	83.24	*	58.98	*
	Wald test for endogeneity χ^2^(1)	4.09	†	86.45	*	61.43	*
	lag 3 years	-0.89 (0.22)	*	-0.20 (1.51)		-0.91 (0.31)	*
	Weak Instrument (Anderson-Rubin Wald test F)	22.22	*	0.02		10.40	*
	Wald test for endogeneity χ^2^(1)	23.59	*	0.08		11.78	*

Note: *: *p*-value < 0.01; †: *p*-value < 0.05

## Discussion

This study established ICOC as a potentially new index to investigate the cost-saving effect using an 8-year database of newly diagnosed diabetes patients. As many medical groups are adopting an integrated team care approach for chronic disease management, this ICOC index serves as a valid and reliable indicator to provide important policy reference in the evidence-based policy making process. Furthermore, we think it is essential to use a 2SLS method to discern the ICOC’s elasticities for outpatient and inpatient costs in order to avoid the problem of endogeneity. This study’s findings are against the common practice of treating COC as exogenous in analyzing inpatient use/costs. The advantage of using 2SLS estimation model is to reduce potential bias caused by endogeneity of the key variable, ICOC. For example, a patient who is health-conscious and complying to the doctor’s orders would likely show higher ICOC whilst maintaining good health. In this case, the cost-saving effect of ICOC and unobserved individual effect (e.g. personal health-consciousness) may not be disentangled. While analyzing the ICOC effect on the outpatient costs with a single OLS equation, the estimated elasticity is -0.07, meaning a 1 % increase in ICOC score would reduce outpatient costs by 0.07 %, a combined effect of ICOC and personal health-consciousness. Hence, we exploited the local prevalence of P4P program as an IV for ICOC, by design, the IV is related to ICOC but not unobserved individual effect, therefore producing a cost-saving effect solely attributed to ICOC. The ICOC elasticity of 1.03 from the 2SlS model signifies a 1 % increase in ICOC score will actually increase outpatient costs by 1.03 %, which is an unbiased estimate of cost-saving effect.

Through IV, we have estimated the average effect of the ICOC on health costs, and the Andersen-Rubin Wald F statistics rejected the hypothesis that average local provider P4P participation rate was a weak instrument. However, in nonexperimental settings, when treatment effects (the impact of ICOC on health costs in this paper) are (likely to be) heterogeneous, it may not be possible to draw inferences about the effect of treatment in the population represented by a particular sample of people [34]. That is, our results are applicable to our study sample, but may not be generalized to all DM population.

Our findings also have shown that an increase in the ICOC score is associated with an increase in outpatient costs and a decrease in inpatient expenses. The net effect is that an increase in the ICOC score is associated with a significant saving effect on the total treatment cost.

The estimated ICOC elasticity of -11.6 for inpatient costs have shown great sensitivity of inpatient costs towards changes in ICOC value, which might be attributable to the nature of the ICOC: (1) ICOC has a small range of value between 0 and 1, therefore a change in the number of outpatient visits may result in only a small percentage change in ICOC. (2) Many patients incurred zero inpatient cost (i.e., no hospitalization), hence a decrease in inpatient costs is likely to result in a substantial change in costs in percentage. As the distribution of inpatient costs has a big spike of zeros and is skewed to the right, the ICOC elasticity estimated from the 2SLS might be biased. In light of recent development in analyzing health care expenditures with generalized linear models (GLM) and two-stage residual inclusion (2SRI), we did a robustness check using 2SRI with GLM to estimate ICOC elasticity for inpatient costs [35, 36]. The results show that the estimated ICOC elasticity is -12.50 (assuming Gamma distribution; results not shown, upon request) which is similar to that of 2SLS, -11.62. Given that the estimated ICOC from 2SRI model is similar to that from the 2SLS model, for the ease of interpretation, we have decided to keep the 2SLS results in this paper.

The estimated ICOC elasticity for total costs is -1.07, which means at the sample average ICOC (around 0.8), a 1 % increase in ICOC score would decrease total costs by 1.07 %. To the best of our knowledge, no study has ever estimated ICOC elasticity. However, quite a few studies have used elasticity to assess the impact of cost-sharing on total spending, and the estimated arc price elasticities range between 0.14–0.43[37]; 0.10–2.56 [38]; and 0.01–0.44 [39]. Except for Cutler et al. (2000), 1 % increase in cost-sharing is likely to result in less than 1 % decrease in total spending. Given the above findings, clinic visits appear to produce cost-saving under the payment system even though the NHI provides clinics with a lower reimbursement rate than hospitals for the same services. When we applied sub-group panel regression analyses to estimate ICOC elasticities for just the clinics data, we found a promising cost-saving effect of ICOC. As shown in Table 4, the ICOC elasticities using 2SLS estimation are all negative for patients who mainly visited clinics. In contrast, the ICOC elasticity for hospital outpatient costs is positive probably because outpatient services in hospitals are likely to exploit expensive diagnostic testing and high-end medical technology.

**Table 4:** ICOC elasticity for sub-samples

		ICOC elasticity					
		OP costs		IP costs		Total costs	
**OLS model**	Clinics only (n = 9,437)	-0.33 (0.007)	*	-1.49 (0.04)	**	-0.52 (0.01)	**
	Hospitals only (n = 16,975)	-0.03 (0.006)	*	-1.68 (0.04)	*	-0.24 (0.007)	*
**2SLS model**	Clinics only	1.03 (0.26)	*	-11.62 (1.93)	*	-1.07 (0.31)	*
	Weak Instrument (Anderson-Rubin Wald test F)	23.3	*	61.46	*	12.54	*
	Wald test for endogeneity χ^2^(1)	30.56	*	46.68	*	5.75	†
	Hospitals only	0.28 (0.20)		-3.21 (1.52)	†	-0.50 (0.28)	
	Weak Instrument (Anderson-Rubin Wald test F)	2.04		4.45	†	3.21	
	Wald test for endogeneity χ^2^(1)	2.43		1.02		0.87	

Note: *: p-value < 0.01; †: p-value < 0.05

Two research constraints bear noting. First, our findings are applicable only to new diabetes patients who could maintain at least 4 visits per year. Second, in some township where there is only one hospital, the average P4P participation rate equals to that provider’s P4P participation decision (zero or 1) and may be subject to endogeneity problem. Nonetheless, as the majority of townships (ranging from 91 to 93 % during the observation period) have more than one provider, the endogeneity issue is likely to be ameliorated.

## 5 Conclusions

This study expands the boundary of continuity of care (COC) to explicitly incorporate the COC effect at the institutional level and then evaluates the relative cost-saving effects of ICOC among outpatient, inpatient, and total treatment costs. Institutional characteristics are likely to have impacts on treatment costs for diabetes patients. Institutional continuity of care (ICOC) is associated with higher outpatient costs but lower inpatient costs. Clearly, ICOC’s impact on total treatment costs depends on the relative magnitude of effects on inpatient vs. outpatient costs. As a result, the cost-saving effect of ICOC appear to be much greater in countries where inpatient care costs are substantially higher than the costs of outpatient care.

## Data Availability

Data abstracted from the National Health Insurance Research Database provided by the National Health Insurance Administration, Ministry of Health and Welfare, was managed by National Health Research Institutes. Restrictions apply to the availability of these data, which were used under license for the current study, and so are not publicly available.

## Appendix

**Appendix 1:** Illustration of ICOC index change with the number of visits

n_1_ (principal provider)	n_2_ (secondary provider)	sum n^2^	N	N-1	ICOC
3	1	10	4	3	0.50
4	1	17	5	4	0.60
3	2	13	5	4	0.40
6	2	40	8	7	0.57
7	2	53	9	8	0.61
6	3	45	9	8	0.50
5	4	41	9	8	0.44
6	4	52	10	9	0.47
5	5	50	10	9	0.44
13	1	170	14	13	0.86
14	1	197	15	14	0.87
13	2	173	15	14	0.75

## Abbreviations

ACO: Accountable care organizations
COC: Continuity of care
DCSI: Diabetes Complications Severity Index
DM: Diabetes mellitus
GLM: Generalized linear model
GP: General practitioner
ICOC: Institutional continuity of care
IDF: International Diabetes Federation
IV: Instrumental variable
NHI: National Health Insurance
OLS: Ordinary least squares
PCMH: Patient-centered medical homes
P4P: Pay-for-performance
2SLS: Two-stage least squares
2SRI: Two-stage residual inclusion
US: United States

## References

[1] International Diabetes Federation. IDF DIABETES ATLAS, Ninth edn. Brussels; 2019.

[2] GBD 2016 Disease and Injury Incidence and Prevalence Collaborators (2017). Global, regional, and national incidence, prevalence, and years lived with disability for 328 diseases and injuries for 195 countries, 1990–2016: a systematic analysis for the Global Burden of Disease Study 2016. Lancet.

[3] GBD 2019 Diseases and Injuries Collaborators (2020). Global burden of 369 diseases and injuries in 204 countries and territories, 1990–2019: a systematic analysis for the Global Burden of Disease Study 2019. Lancet.

[4] Kerrissey MJ, Clark JR, Friedberg MW, Jiang W, Fryer AK, Frean M, Shortell SM, Ramsay PP, Casalino LP, Singer SJ (2017). Medical Group Structural Integration May Not Ensure That Care Is Integrated, From The Patient’s Perspective. Health Aff (Millwood).

[5] Accountable Care Organizations (ACOs) [https://www.cms.gov/Medicare/Medicare-Fee-for-Service-Payment/ACO/index].

[6] Transforming the organization and delivery of primary care. https://pcmh.ahrq.gov/.

[7] Mendel P, Chen EK, Green HD, Armstrong C, Timbie JW, Kress AM, Friedberg MW, Kahn KL (2018). Pathways to Medical Home Recognition: A Qualitative Comparative Analysis of the PCMH Transformation Process. Health Serv Res.

[8] Afendulis CC, Hatfield LA, Landon BE, Gruber J, Landrum MB, Mechanic RE, Zinner DE, Chernew ME (2017). Early Impact Of CareFirst’s Patient-Centered Medical Home With Strong Financial Incentives. Health Aff (Millwood).

[9] Bongaerts BW, Mussig K, Wens J, Lang C, Schwarz P, Roden M, Rathmann W. Effectiveness of chronic care models for the management of type 2 diabetes mellitus in Europe: a systematic review and meta-analysis. BMJ Open. 2017;7(3). 10.1136/bmjopen-2016-013076 PMC5372084 28320788

[10] For first time, physician practice owners are not the majority [https://www.ama-assn.org/practice-management/economics/first-time-physician-practice-owners-are-not-majority].

[11] Sessums LL, Conway PH (2017). Saving Primary Care. JAMA Intern Med.

[12] Bujold E (2017). The Impending Death of the Patient-Centered Medical Home. JAMA Intern Med.

[13] McWhinney I (1997). A textbook of family medicine.

[14] Kao YH, Lin WT, Chen WH, Wu SC, Tseng TS (2019). Continuity of outpatient care and avoidable hospitalization: a systematic review. Am J Manag Care.

[15] Schwarz D, Hirschhorn LR, Kim JH, Ratcliffe HL, Bitton A (2019). Continuity in primary care: a critical but neglected component for achieving high-quality universal health coverage. BMJ Glob Health.

[16] Flach SD, McCoy KD, Vaughn TE, Ward MM, Bootsmiller BJ, Doebbeling BN (2004). Does patient-centered care improve provision of preventive services?. J Gen Intern Med.

[17] Guo JY, Chou YJ, Pu C (2017). Effect of Continuity of Care on Drug-Drug Interactions. Med Care.

[18] Chen CC, Tseng CH, Cheng SH (2013). Continuity of care, medication adherence, and health care outcomes among patients with newly diagnosed type 2 diabetes: a longitudinal analysis. Medical care.

[19] Worrall G, Knight J (2011). Continuity of care is good for elderly people with diabetes: retrospective cohort study of mortality and hospitalization. Can Fam Physician.

[20] Pan CC, Kung PT, Chiu LT, Liao YP, Tsai WC (2017). Patients with diabetes in pay-for-performance programs have better physician continuity of care and survival. American Journal of Managed Care.

[21] Lubloy A, Kereszturi JL, Benedek G (2016). Formal Professional Relationships between General Practitioners and Specialists in Shared Care: Possible Associations with Patient Health and Pharmacy Costs. Applied Health Economics Health Policy.

[22] Gulliford M, Cowie L, Morgan M (2011). Relational and management continuity survey in patients with multiple long-term conditions. J Health Serv Res Policy.

[23] Waibel S, Henao D, Aller MB, Vargas I, Vazquez ML (2012). What do we know about patients’ perceptions of continuity of care? A meta-synthesis of qualitative studies. Int J Qual Health Care.

[24] Pu C, Chou YJ (2016). The impact of continuity of care on emergency room use in a health care system without referral management: an instrumental variable approach. Ann Epidemiol.

[25] Jee SH, Cabana MD (2006). Indices for continuity of care: a systematic review of the literature. Medical Care Research Review.

[26] National Health Insurance Administration (2019). National Health Insurance Annual Statistical Report 2018.

[27] International Diabetes Federation (2017). **IDF DIABETES ATLAS**. In., Eighth edition edn.

[28] National Health Insurance Pay-for-Performance Program for Diabetes [in Chinese]. [https://www.nhi.gov.tw/Resource/webdata/29807_1_Y104-4_V4.pdf].

[29] Contoyannis P, Hurley J, Grootendorst P, Jeon S-H, Tamblyn R (2005). Estimating the Price Elasticity of Expenditure for Prescription Drugs in the Presence of Non-linear Price Schedules: An Illustration from Quebec, Canada. Health Econ.

[30] Selby JV, Karter AJ, Ackerson LM, Ferrara A, Liu J (2001). Developing a prediction rule from automated clinical databases to identify high-risk patients in a large population with diabetes. Diabetes Care.

[31] Barker I, Steventon A, Deeny SR (2017). Association between continuity of care in general practice and hospital admissions for ambulatory care sensitive conditions: cross sectional study of routinely collected, person level data. BMJ.

[32] Tung YC, Chang GM, Chen YH (2009). Associations of physician volume and weekend admissions with ischemic stroke outcome in Taiwan: a nationwide population-based study. Med Care.

[33] Wooldridge JM (2010). Econometric Analysis of Cross Section and Panel Data.

[34] Harris KM, Remler DK (1998). Who is the marginal patient? Understanding instrumental variables estimates of treatment effects. Health Serv Res.

[35] Deb P, Norton EC (2018). Modeling Health Care Expenditures and Use. Annu Rev Public Health.

[36] Terza JV (2018). Two-Stage Residual Inclusion Estimation in Health Services Research and Health Economics. Health Serv Res.

[37] Manning WG, Newhouse JP, Duan N, Leibowitz BKE. A: Health Insurance and the Demand for Medical Care: Evidence from a Randomized Experiment. Am Econ Rev. 1987;77(3):251–77. 10284091

[38] Cutler DM, Zeckhauser RJ: The Anatomy of Health Insurance. In: Handbook of Heatlh Economics Volume 1A, edn. Edited by Culyer AJ, Newhouse JP. Amsterdam: Elsevier Science B.V.; 2000: 564–643.

[39] Ellis RP, Martins B, Zhu W (2017). Health Care Demand Elasticities by Type of Service. Journal of Health Economics.

